# Soft Tissue Infection of the Forearm With Scedosporium apiospermum Complex and Neisseria spp. Following a Dog Bite

**DOI:** 10.7759/cureus.14140

**Published:** 2021-03-27

**Authors:** Vidya S Kollu, Jena Auerbach, Alaina S Ritter

**Affiliations:** 1 Department of Infectious Diseases and Global Medicine, College of Medicine, University of Florida, Gainesville, USA; 2 Department of Pathology, Immunology and Laboratory Medicine, College of Medicine, University of Florida, Gainesville, USA

**Keywords:** scedosporium apiospermum, dog bite, neisseria spp, splendore-hoeppli phenomenon

## Abstract

We report a case of a complex skin and soft tissue infection caused by *Scedosporium apiospermum* complex and *Neisseria* *spp.* following a dog bite. While Neisseria skin and soft tissue infections after dog bites have been reported, only one case of subsequent infection caused by *Scedosporium spp.* has been noted in the literature. To the best of our knowledge, this is the first reported case of coinfection of these particular organisms following a dog bite.

## Introduction

Dog bite injuries are a common public health problem. According to a survey conducted between 2001 and 2003, the estimated incidence of dog bites in the United States is 4.5 million per year, and 885,000 require medical attention [1]. Dog bites are usually polymicrobial, and commonly isolated pathogens include *Pasteurella canis*, Staphylococci, Streptococci, Moraxella, *Neisseria spp*.,Fusobacterium, Porphyromonas, Bacteroides, and Prevotella [2]. Fungal pathogens are uncommon, and there is only one reported case of *Pseudallescheria boydii* (now known as *Scedosporium boydii*) causing tenosynovitis following a dog bite [3]. Our report describes a complex skin and soft tissue infection caused by *Scedosporium apiospermum *complex and *Neisseria spp*. in the setting of a dog bite.

## Case presentation

**Figure 1:**
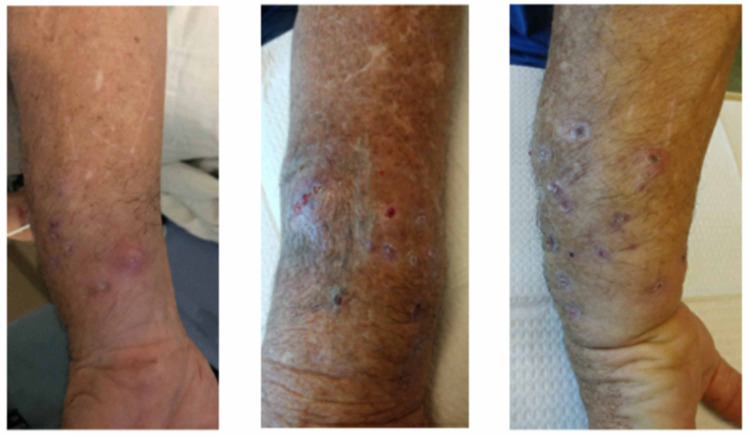
Left forearm lesions prior to treatment

A 58-year-old man with a past medical history of hypertension, spinal stenosis, and depression presented to the Infectious Diseases clinic with complaints of left forearm swelling and pain. He reported that five years prior to the presentation, he broke up a fight between two dogs and was bitten by both dogs on his left forearm. He returned home, washed the wounds with tap water, and used superglue to close the puncture wounds. He did not seek medical attention. Several months later, he reportedly developed a pustule on the arm that ruptured and then resolved. He subsequently noted pustules periodically arising on the forearm, draining purulent material, and then scabbing. Over the next few years, he developed increasing induration and nodularity of the forearm with worsening pain and paresthesias of the fingers. He was never evaluated by a physician for these symptoms. Several months prior to his presentation, he was handcuffed, which resulted in pressure being placed on his left wrist. His left arm subsequently became very swollen and painful, which prompted him to present to the emergency department for evaluation. On evaluation, he was afebrile. His left arm was noted to be exquisitely tender with woody induration and scabbing (Figure 1).

**Figure 2:**
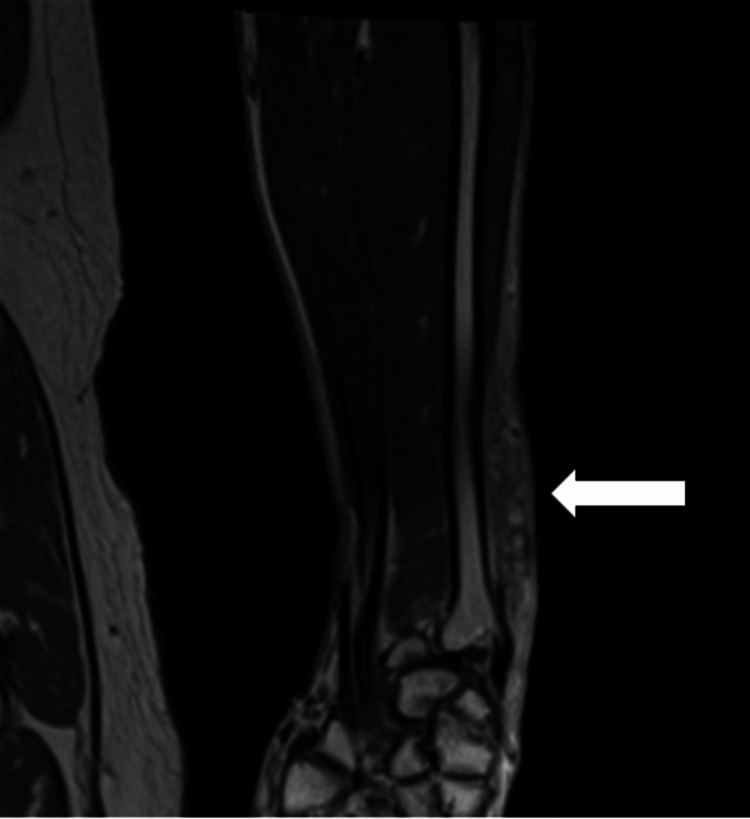
MRI showing inflammatory changes and edema in the soft tissues of the left forearm

Labs showed a normal white blood cell count (7.3 x 10^3^ cells/mm^3^, normal range: 4.0-10.0), normal hemoglobin (13.7 g/dL, normal range: 13.0-16.5), and normal creatinine (0.9 mg/dL, normal range: 0.38-1.02). His inflammatory markers were elevated with a high sensitivity C-reactive protein of 7.01 mg/L (normal range: 0.0-4.9) and a sedimentation rate of 36 mm/hr (normal range: 0.0-20). He also had reduced sensation in the fingers of the left hand. A computerized tomography (CT) scan was obtained that showed soft-tissue edema in the hypodermis of the left forearm and the wrist compatible with cellulitis as well as focal hypoattenuation with extension into the dorsal muscle compartment that was suspicious for suppuration. Magnetic resonance imaging (MRI) done for further evaluation did not show any evidence of an abscess/drainable fluid collection or osteomyelitis, but it confirmed inflammatory changes and edema in the soft tissues (Figure 2).

**Figure 3:**
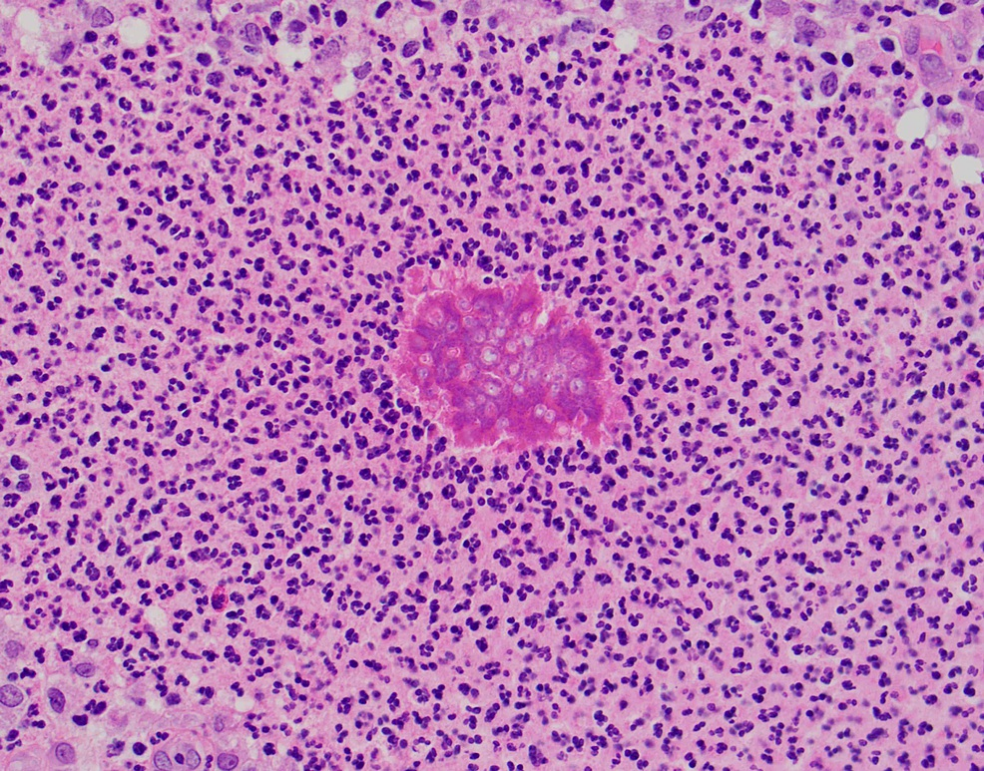
Splendore-Hoeppli phenomenon within the abscess

He was discharged home on clindamycin 300 mg PO every six hours for seven days. At his orthopedic follow-up appointment several weeks later, he reported no improvement in the swelling or pain. He subsequently underwent an open biopsy of several lesions on the left forearm. Necrotic tissue and dense scar tissue were encountered but no purulence was noted. All the necrotic and scar tissue was excised. He was prescribed topical miconazole post-operatively. Pathology showed pseudoepitheliomatous hyperplasia, chronic inflammation, microabscesses, focal giant cells, and scar tissue. Gomori Methenamine Silver stain and Periodic Acid-Schiff stain were negative for fungal microorganisms. However, within the abscess, there was noted to be asteroid-like brightly eosinophilic material (Splendore-Hoeppli phenomenon) with focal spherical forms suggestive of fungal microorganisms (Figure 3).

Cultures grew *Neisseria spp.* and *S. apiospermum* complex. Scedosporiumspeciation was confirmed using DNA sequencing (sensitivities in Table 1).

**Table 1:** Susceptibilities of Scedosporium apiospermum complex

Antibiotic	MIC
Amphotericin B	1 µg/mL
Anidulafungin	4 µg/mL
Caspofungin	1 µg/mL
Fluconazole	16 µg/mL
Isavuconazole	8 µg/mL
Itraconazole	8 µg/mL
Micafungin	0.06 µg/mL
Posaconazole	1 µg/mL
Terbinafine	>2 µg/mL
Voriconazole	1 µg/mL

Acid-fast bacteria cultures were negative at 42 days.

**Figure 4:**
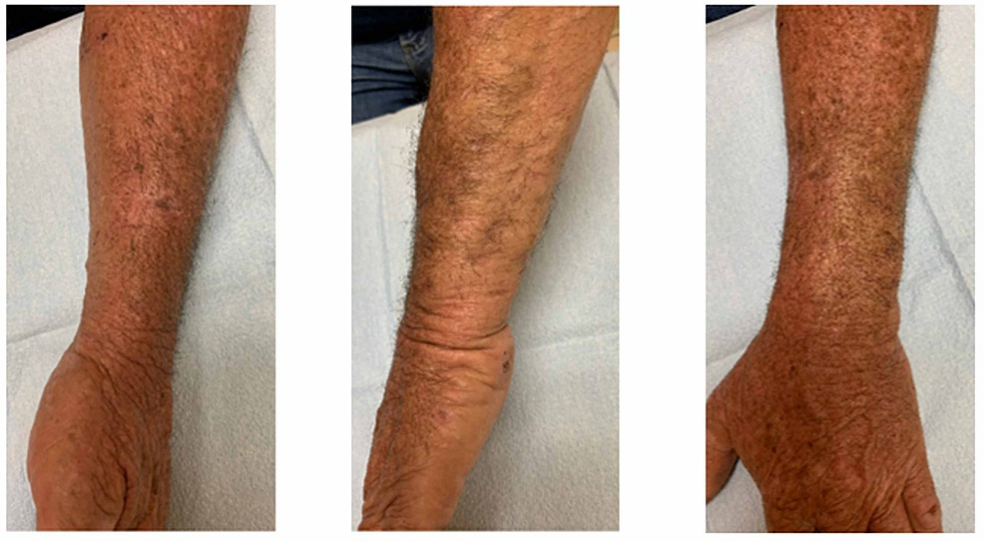
Left forearm lesions after six months of treatment

The patient was started on amoxicillin/clavulanate 875 mg/125 mg by mouth twice a day to treat the *Neisseria spp*. and voriconazole 200 mg by mouth twice daily to treat the *S. apiospermum* complex. After completing six weeks of amoxicillin/clavulanate and six months of voriconazole therapy, he experienced significant improvement in his left forearm swelling, pain, and skin lesions (Figure 4).

## Discussion

To date, there is only one reported case of *P. boydii* (now known as *S. boydii*) causing tenosynovitis following a dog bite and there is no prior reported case of coinfection with *Neisseria spp*. and *S. apiospermum* complex [3]. *S. apiospermum* complex includes *S. apiospermum*, *S. boydii* (previously called *P. boydii*), *S. angustum*, *S. ellipsoideum*, and *S. fusarium* [4]. These fungi are present in freshwater, especially water that is stagnant and polluted, as well as in soil [5]. *Scedosporium spp*. are known to cause a wide variety of clinical infections, including mycetomas and infections of the lungs, sinuses, eye, brain, bone, joints, and heart valves [6]. These infections are usually acquired through inhalation or traumatic inoculation through the skin/epithelium [5]. Usually, the disease is localized in immunocompetent individuals but can become invasive/disseminated in immunocompromised individuals [7]. The mortality rate is often high in immunocompromised individuals, especially in the setting of pulmonary (up to 57.2%) and central nervous system (up to 76%) involvement [5,7]. In immunocompetent individuals, such as our patient, it can sometimes take months to years before symptoms manifest [8].

Scedosporium infection can be diagnosed by culture, direct microscopy with histochemical staining, histopathology, and DNA sequencing. It is typically diagnosed based on culture from the affected site and identified by DNA sequencing [6]. *Scedosporium spp*. may be resistant to amphotericin B and can break through therapy. In vitro susceptibilities to azoles are variable, and there can be reduced susceptibility to echinocandins [9,10]. Voriconazole is considered the drug of choice as it typically has the best in vitro activity and clinical outcomes. In isolates where voriconazole has less in vitro activity, there have been reports of successful treatment with voriconazole in combination with terbinafine or an echinocandin [11]. In a study of 107 patients treated with voriconazole, the majority of those who were successfully treated received between 13 and 799 days of treatment with a median treatment duration of 180 days. A high rate of success was noted in patients with no immunocompromising conditions [11]. Our patient did not have an underlying immunocompromising condition and improved significantly on monotherapy with voriconazole. Terbinafine was not added as there was relative resistance noted to terbinafine on sensitivity analysis. He will continue voriconazole therapy past six months, with the ultimate duration depending on clinical response.

*Neisseria spp*. have been described as pathogens from dog bites as early as 1974. The group includes *N. weaveri*, *N. animaloris*, and *N. Zoodegmatis* [12,13]. These species are considered to be normal oral flora in dogs, cats, and rodents. In humans, they primarily cause purulent wound infections but are also reported to cause pulmonary infections, chronic otitis media, endophthalmitis, tenosynovitis, and bacteremia [12,13]. The specific strain of Neisseria was not identified in our patient. Most strains are susceptible to amoxicillin-clavulanate [14]. Our patient was treated with six weeks of amoxicillin-clavulanate, given his complex skin and soft tissue infection, and experienced appropriate clinical improvement.

## Conclusions

We report a unique case of a *S. apiospermum* complex and *Neisseria spp. *coinfection after a dog bite. After six months of antifungal/antibiotic treatment, the patient experienced significant improvement of his left forearm complex skin and soft tissue infection. Thus, it is reasonable for physicians to consider these organisms as possible pathogens in patients with infections following a dog bite. Since *Scedosporium spp*. may have variable susceptibilities to amphotericin B, echinocandins, and azoles, sensitivity data can help guide therapy. Voriconazole is typically the drug of choice, however, given its in vitro activity.
